# Polymorphisms in the Gene Encoding Caspase 8 May Predict the Response to First-Line Platinum-Based Chemotherapy in Locally Advanced or Advanced Non-Small-Cell Lung Cancer

**DOI:** 10.3390/jcm10051126

**Published:** 2021-03-08

**Authors:** Michał Szczyrek, Radosław Mlak, Aneta Szudy-Szczyrek, Karolina Kędziora, Teresa Małecka-Massalska, Paweł Krawczyk, Janusz Milanowski

**Affiliations:** 1 Department of Pneumonology, Oncology and Allergology, Medical University of Lublin, 20-090 Lublin, Poland; pawel.krawczyk@umlub.pl (P.K.); janusz.milanowski@umlub.pl (J.M.); 2 Department of Human Physiology, Medical University of Lublin, 20-080 Lublin, Poland; radoslawmlak@umlub.pl (R.M.); teresa.malecka-massalska@umlub.pl (T.M.-M.); 3 Department of Haematooncology and Bone Marrow Transplantation, Medical University of Lublin, 20-081 Lublin, Poland; aneta.szudy-szczyrek@umlub.pl; 4 Collegium Medicum, University of Zielona Góra, 64-064 Zielona Góra, Poland; karolina0kedziora@gmail.com

**Keywords:** caspase 8, *CASP-8*, NSCLC, platinum, polymorphism

## Abstract

Caspase 8 is a protein involved in the process of cell apoptosis, which may affect the efficacy of anti-cancer treatment. The aim of our study was to determine the impact of polymorphisms in the *CASP-8* gene encoding caspase 8 on the prognosis in non-small-cell lung cancer (NSCLC). The study involved 99 patients with newly diagnosed locally advanced or metastatic NSCLC treated with platinum-based chemotherapy. The presence of the GG genotype was associated with distant metastases, smoking, and a family history of cancer. The higher risk of early progression was associated with weight loss and the *CASP-8* genotype (GG vs. AG or AA: 20.51% vs. 2.86%). The higher risk of progression-free survival (PFS) shortening was associated with a higher stage of disease (hazard ratio (HR) = 2.50, 95% CI: 1.61–3.89, *p* < 0.0001), distant metastases (HR = 2.30, 95% CI: 1.42–3.72, *p* = 0.0016), and the GG genotype (HR = 1.68, 95% CI: 1.10–2.57, *p* = 0.0152). The influence of the GG genotype on the PFS was confirmed in a multivariate analysis (HR = 1.80, 95% CI: 1.06–3.05, *p* = 0.0317). We did not confirm the influence of *CASP-8* genotypes on the overall survival (OS).

## 1. Introduction

Non-small-cell lung cancer (NSCLC) is the most common pulmonary malignancy and constitutes around 80% of lung cancers. It is often diagnosed in late stages of the disease, which are not eligible for surgical treatment and require chemotherapy, chemoradiotherapy, immunotherapy, chemoimmunotherapy, and/or molecularly targeted therapies. First-line chemotherapy with platinum-based regimens is effective only in 30–40% of patients, with no established molecular predictive factors for the treatment.

Caspases play a central role in the process of cell apoptosis induced by Fas ligand (FasL) and various other apoptotic stimuli. Evidence suggests that caspases are inhibited in NSCLC, which may affect the efficacy of treatment [1]. Caspase 8, a protein encoded by the *CASP-8* gene, is a member of the cysteine–aspartic acid protease (caspase) family. It was proven that platinum-based chemotherapy triggers apoptosis, creating intra- and interstrand cross-links in DNA and inhibiting proper DNA replication. DNA damage leads to the activation of the caspase cascade. Therefore, caspase downregulation can lead to cisplatin resistance in the mechanism of delaying apoptosis and extending the time for DNA repair [2,3].

The aim of this study was to determine whether specific genotypes of the *CASP-8* gene affect the efficacy of platinum-based chemotherapy in patients with advanced NSCLC.

## 2. Materials and Methods

The study group comprised 99 Caucasian patients with newly diagnosed locally advanced or metastatic NSCLC enrolled between 7 January 2016 and 20 April 2017. The staging of disease was determined according to the Tumor Nodes Metastases (TNM) classification (VII edition), and the response to treatment was evaluated according to Response Evaluation Criteria in Solid Tumors (RECIST) version 1.1. The performance status of patients was assessed on the Eastern Cooperative Oncology Group/World Health Organization (ECOG/WHO) scale. All patients received first-line chemotherapy with platinum-based doublet regimens. Detailed characteristics of the patients are presented in Table 1.

First, 5 mL of whole blood was drawn from all participants at the time of enrollment, prior to chemotherapy, and stored at −80 °C until laboratory analyses. The DNA Blood Mini Kit (Qiagen, Toronto, Ont., Canada) was used to isolate DNA. The quality and quantity of DNA were assessed using the NanoDrop Lite Spectrophotometer (Thermo Fisher Scientific, Bedford, MA, USA). The evaluation of single-nucleotide polymorphisms (SNPs) of the *CASP-8* gene was performed using a real-time PCR method with allelic discrimination software. The Genotyping Master Mix and TaqMan probes (Applied Biosystems, USA) specific for the studied SNPs (Thermo Fisher Scientific, Bedford, MA, USA) were used for DNA amplification according to the manufacturer’s protocol in an RT7500 real-time PCR device (Applied Biosystems, Carlsbad, CA, USA). All tests were run in triplicate. The study was performed based on the approval of the institutional research committee (Bioethical Commission of Medical University of Lublin; consent reference number KE-0254/219/2015), in accordance with the 1964 Declaration of Helsinki and its later amendments.

All statistical analyses were performed using MedCalc 15.8 (MedCalc Software, Ostend, Belgium). Data were expressed as a percentage (for the categorized variable), median, and range (for continuous variables). The distribution of individual *CASP-8* gene genotypes was estimated in groups of patients with continuous variables above and below the median (applies to age, pack-years, weight, body mass index (BMI), weight loss, time from diagnosis to treatment). We considered *p*-values below 0.05 to be statistically significant. The risk of early progression was assessed with the use of the odds ratio (OR) test. The analysis of progression-free survival (PFS), time to progression (TTP), and overall survival (OS) was carried out using the Kaplan–Meier estimation method, with calculation of the hazard ratio (HR) and 95% confidence interval (CI). Univariate analysis was performed with the use of the Kaplan–Meier estimation method (log-rank), whereas Cox logistic regression models were used in multivariate analysis with statistically significant factors from univariate analysis (α < 0.05) as included variables.

## 3. Results

### 3.1. Distribution of CASP-8 Genotypes and Their Influence on the Risk of Early Progression

The study group was dominated by men (76.77%). The median age of patients was 66.5 years (range: 44 to 83 years). Stages IIIB and IV of NSCLC were diagnosed in 64.65% of patients and distant metastases in 56.57% of patients. In addition, 58.59% of patients had a very good or good performance status (PS = 0 or 1). The most common histological diagnosis was squamous cell carcinoma (SCC; 52.53% of patients) and adenocarcinoma (AC; 40.40% of patients). The most commonly used treatment regimens were cisplatin with vinorelbine (60.60%), cisplatin with pemetrexed (23.23%), and cisplatin with gemcitabine (16.16%). The detailed demographic and clinical characteristics of the study group are given in Table 1.

Among the demographic and clinical factors, only distant metastases, smoking status, and a family history of malignancy (including lung cancer) were significantly related to the incidence of individual *CASP-8* gene genotypes. The GG genotype was significantly more common in patients with distant metastases (60%), smokers (56.52%), patients with a family history of malignancy (60.78%), and patients with a history of lung cancer in family members (65.38%). Detailed data on the impact of demographic and clinical factors on the distribution of *CASP-8* genotypes are presented in Table 2.

The following demographic, clinical, and genetic factors were significantly associated with a higher risk of disease progression during the first assessment (after two cycles): weight loss (24% vs. 6.52%, OR = 4.84, 95 % CI: 1.09–21.39, *p* = 0.0374) and AG or AA genotypes of the *CASP-8* gene (GG vs. AG or AA: 20.51% vs. 2.86%, OR = 8.77, 95% CI: 1.04–74.21, *p* = 0.0462). Detailed data on the impact of demographic, clinical, and genetic factors on the risk of disease progression (assessed after two cycles) in the study group are given in Table 3.

A significantly higher risk of disease progression during the second assessment (after four cycles) was associated with weight loss (46.67% vs. 2.86%, OR = 29.75, 95% CI: 3.19–277.33, *p* = 0.0029), longer time from diagnosis to treatment start (28% vs. 4%, OR = 9.33, 95% CI: 1.05–82.78, *p* = 0.0449), and AG or AA genotypes of the *CASP-8* gene (GG vs. AG or AA: 29.17% vs. 3.85%, OR = 10.29, 95% CI: 1.16–91.43, *p* = 0.0364). Detailed data on the impact of demographic, clinical, and genetic factors on the risk of disease progression assessed after four cycles are given in Table 4.

### 3.2. Univariate Survival Analysis

**Figure 1:**
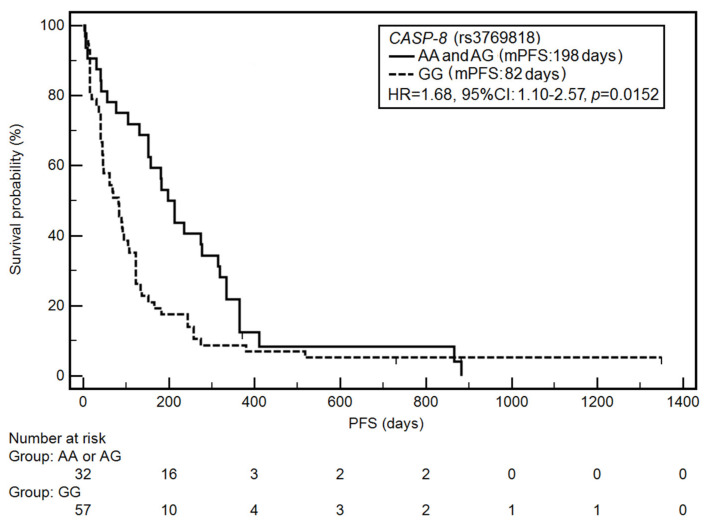
Kaplan–Meier curves illustrating progression-free survival (PFS) differences between genotypes AA and AG, GG of the *CASP-8* gene (rs3769818).

Among the studied demographic, clinical, and genetic factors, the higher risk of PFS shortening was associated with a higher stage of disease (HR = 2.50, 95% CI: 1.61–3.89, *p* < 0.0001), a lower number of chemotherapy cycles (HR = 4.24, 95% CI: 2.07–8.72, *p* <0.0001), the presence of distant metastases (HR = 2.30, 95% CI: 1.42–3.72, *p* = 0.0016), and the GG genotype of the *CASP-8* gene (HR = 1.68, 95% CI: 1.10–2.57, *p* = 0.0152) (Figure 1).

**Figure 2:**
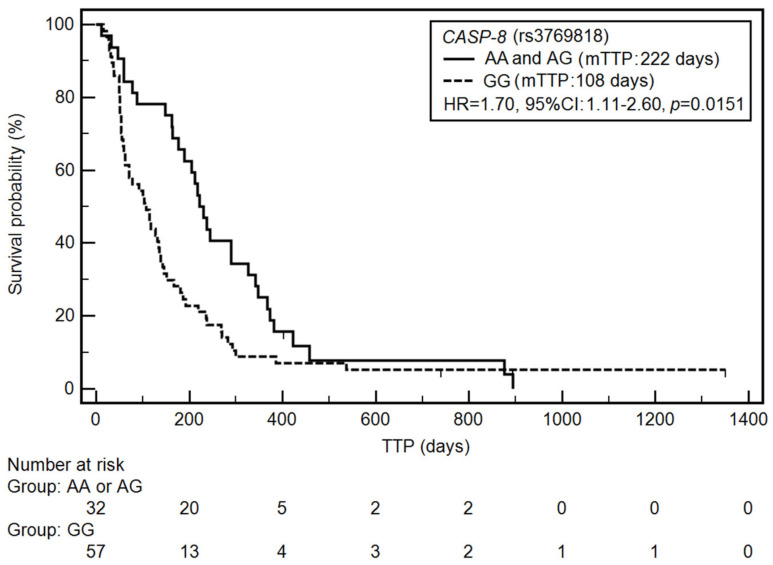
Kaplan–Meier curves illustrating time-to-progression (TTP) differences between genotypes AA and AG, GG of the *CASP-8* gene (rs3769818).

In contrast, the higher risk of TTP shortening was associated with a higher stage of disease (HR = 2.56, 95% CI: 1.67–3.92, *p* < 0.0001), a longer smoking period (HR = 1.54, 95% CI: 1.00–2.36, *p* = 0.0408), the use of cisplatin and pemetrexed in first-line chemotherapy (HR = 1.72, 95% CI: 0.97–3.03, *p* = 0.0240), a lower number of cycles (HR = 3.80, 95% CI: 1.90–7.59, *p* < 0.0001), the presence of distant metastases (HR = 2.36, 95% CI: 1.46–3.82, *p* = 0.0013), the GG genotype of the *CASP-8* gene (HR = 1.70, 95% CI: 1.11–2.60, *p* = 0.0151) (Figure 2). The use of cisplatin and vinorelbine as the first line of treatment was associated with a significant reduction in the risk of TTP shortening (HR = 0.64, 95% CI: 0.40–1.02, *p* = 0.0380).

Similarly, a higher risk of OS shortening was associated with a higher stage of disease (HR = 2.01, 95% CI: 1.30–3.09, *p* = 0.0021), a lower number of cycles (HR = 2.39, 95% CI: 1.31–4.36, *p* = 0.0005), the presence of distant metastases (HR = 2.28, 95% CI: 1.40–3.73, *p* = 0.0026), and higher weight loss (HR = 1.60, 95% CI: 0.95–2.69, *p* = 0.0428).

Progression-free survival, time to progression, and overall survival according to demographic, clinical, and genetic factors are demonstrated in Table 5.

### 3.3. Multivariate Survival Analysis

Multivariate analysis showed that independent factors associated with PFS shortening included a higher stage of disease (HR = 2.56, 95% CI: 1.43–7.76, *p* = 0.0016), the presence of distant metastases (HR = 3.16, 95% CI: 1.09–9.15, *p* = 0.0353), and the GG genotype of the *CASP-8* gene (HR = 1.80, 95% CI: 1.06–3.05, *p* = 0.0317). In turn, in the case of the TTP, independent factors associated with its shortening were a higher stage of disease (HR = 3.87, 95% CI: 1.28–11.70, *p* = 0.0167) and histopathological diagnosis of squamous cell carcinoma (HR = 2.44, 95% CI: 1.35–4.35, *p* = 0.0028). On the other hand, independent prognostic factors associated with OS shortening included a higher stage of disease (HR = 2.94, 95% CI: 1.64–5.26, *p* = 0.0002), a lower BMI (HR = 1.72, 95% CI: 1.01–2.95, *p* = 0.0474), and unintentional weight loss (HR = 1.95, 95% CI: 1.11–3.44, *p* = 0.0202). Cox’s logistic regression analysis for progression-free survival, time to progression, and overall survival is presented in Table 6.

## 4. Discussion

The molecular origin of lung cancer stems from the accumulation of many genetic and epigenetic changes that occur in the cells over a long period of time [4]. Genome instability is a condition that occurs at the beginning of a neoplastic process and leads to weakening of the DNA structure and its susceptibility to mutations [5]. Disorders include abnormalities in cell cycle regulation (mutations of proto-oncogenes and suppressor genes), DNA repair errors, increased expression of growth and angiogenesis factors, avoidance of apoptosis (mutations of anti- and pro-apoptotic genes), increased telomerase activity, tissue invasion, and metastasis [6]. The search for molecular markers associated with susceptibility to cancer, as well as the response to treatment, is one of the foundations of future lung cancer therapy.

Caspase 8 is a cysteinyl protease that, as one of the initiating proteins, is a critical factor for the activation of the external apoptotic pathway [7]. Procaspase 8, a caspase initiator present as an inactive monomer, is activated in the process of dimerization and then by interaction with many factors, such as cell death receptors or Fas-associated death domain (FADD) [8,9]. Another function of caspase 8 is to regulate inflammatory processes. The protein has been shown to mediate the initiation and activation of the canonical and non-canonical NLRP3 receptor (NLR family, pyrin domain-containing 3) of the inflammasome [10].

Caspase 8 activation induces inflammasome-dependent release of interleukin-1β (IL-1β) from macrophages. It inhibits the receptor-initiating protein (RIP3) kinase, which is involved in the process of necrosis and plays a dominant role in the progression of apoptosis [11,12]. The protein mediates the regulation of T cell proliferation, leading to activation of the NF-κB and mitogen-activated protein kinase (MAPK) pathways. Caspase 8 can therefore be classified as both apoptotic, non-inflammatory and pro-inflammatory along with caspase 1 and caspase 4 (known as caspase 11) [13,14]. Apoptosis and the process of inflammation are two phenomena closely associated with cancerogenesis [14,15]. Inactivation or disturbance of caspase 8 synthesis may cause immortality of damaged cells, which is observed both at the stage of tumor formation and at the stage of progression [16,17,18].

Soung et al. proved the relationship between the inactivated, mutated *CASP8* gene and the development of breast, stomach, and lung cancer [16]. It has also been confirmed that disturbances in *CASP8* expression may be associated with the development of resistance to anti-cancer therapy [19,20].

The role of caspase 8 in the pathogenesis of lung cancer is unclear. Shivapurkar et al. demonstrated that caspase 8 was inactive in small-cell lung cancer (SCLC) patients, and its absence was associated with cancer cell chemoresistance by stimulation of tumor necrosis factor-related apoptosis-inducing ligand (TRAIL) [21]. Riley et al. found that pro-caspase 8 is overexpressed in NSCLC patients and is a marker of poor prognosis [22]. In the work of Terlizzi et al., the activity of caspase 8 protein on human NSCLC cells and on a mouse model of lung cancer was assessed. Pharmacological inhibition of caspase 8 has been shown to reduce tumor growth by reducing the release of pro-inflammatory cytokines (IL-6, TNF-α, IL-18, IL-1α, IL-33), decreased recruitment of innate suppressor cells, and higher cellular FLICE-like inhibitors protein (c-FLIP) expression, which determines the progression of lung cancer [14].

The aim of our study was to analyze the relationship between the polymorphic variants of the *CASP-8* gene (rs3769818) and selected demographic and clinical characteristics as well as treatment outcomes in patients diagnosed with NSCLC who underwent chemotherapy with platinum derivatives. Our study was limited by a small sample size; however, it was planned as a pilot project for further research. The human *CASP-8* gene is located on chromosome 2q33–q34, it has 11 exons [12], and it is highly polymorphic with over 474 single-nucleotide polymorphisms (SNPs) according to the dbSNP database [23]. The relationship between *CASP-8* gene polymorphisms and the pathogenesis of many cancers, including lung cancer, is well documented [24,25].

Son et al. demonstrated that carriers of the *CASP-8* IVS12-19 GG genotype have a significantly higher risk of developing small-cell lung carcinoma compared to carriers of the IVS12–19 AA and IVS12–19 GA genotypes [24]. Ulybina et al. analyzed the influence of 19 polymorphisms in genes coding apoptotic proteins, including *CASP-8*, on the risk of lung cancer. They recruited 111 patients with a clear susceptibility to falling ill, i.e., non-smokers or people smoking sporadically at a young age, and 110 elderly patients after many years of intensive smoking.

Among the genotypes possibly associated with lung cancer risk were Val318Leu of the *CASP-5* gene (OR = 2.47, 95% CI: 1.07–5.69, *p* = 0.03), Lys441Arg of the DR4 gene (OR = 1.89, 95% CI: 1.05–3.40, *p* = 0.03), and His302Asp for *CASP-8* (OR = 2.26, 95% CI: 1.18–4.31, *p* = 0.02) [26]. Hart et al. analyzed 11 functional polymorphisms in nine genes in 882 people from the Norwegian population. The authors showed that the combination of three polymorphisms in the *CASP-8* gene, matrix metalloproteinase-1, selenium and S1 protein, and interleukin-10, was associated with an approximately twofold-higher risk of NSCLC (OR = 2.06, 95% CI: 1.19–3.47), while in people with four genotypes, the risk was estimated to be 4.62 times higher (OR = 4.62, 95% CI: 1.69–12.63) [25].

In the conducted study, we observed that carriers of the GG rs3769818 genotype of *CASP-8* were more often smokers (*p* < 0.0001) and those with a history of cancer in the family (any neoplasm: *p* = 0.0003; lung cancer: *p* = 0.0157). The obtained results suggest that the polymorphic variability of the *CASP-8* gene may therefore determine the NSCLC phenotype.

The relationship between genetic predisposition and smoking behavior is believed to be of key importance in the development of lung cancer. Getting to know them can potentially contribute to the assessment of lung cancer risk and prevention of the disease [27,28,29]. Gene–environment interactions explain the so-called heritability of lung cancer [30].

Genome-wide association studies (GWAS), conducted so far, have identified certain mutations associated with lung cancer. The *CHRNA5*, *CHRNA3*, and *CHRNB4* genes in 15q25; *TERT* in 5p15; the human leukocyte antigens (HLA) region in 6p21; and *TP63* at 3q28 were recognized as susceptibility genes [31,32,33,34,35,36,37,38]. In contrast, most of the identified shared variants had a relatively low genetic effect (odds ratio < 1.5), which may be a fraction of the heredity of lung cancer. Zhang et al., using GWAS, found two SNPs, rs1316298 in the *GNG2* gene and rs4589502 in the *AC110048.2* gene, significantly related to the smoking status in patients with lung cancer (OR = 0.71, *p* = 6.73 × 10^−6^ and OR = 1.55, *p* = 3.84 × 10^−6^). The study group consisted of 3865 lung cancer patients and 4566 healthy people from the Chinese Han population [27].

The largest study of the interaction of SNPs and smoking in lung cancer in the Caucasian population was carried out by Li et al. They analyzed the entire genome in a group of 13,336 NSCLC patients and 13,970 controls. They identified further SNPs, rs6441286 in the *IL12A-AS1* gene and rs17723637 in the *ZNF462* gene, associated with lung cancer risk (OR = 1.24, *p* = 6.96 × 10^–7^ and OR = 1.37, *p* = 3.49 × 10^–7^, respectively). The presence of the rs4751674 polymorphic variant significantly affected the risk of squamous cell carcinoma of the lung (OR = 0.58, *p* = 8.12 × 10^–7^) [39].

To verify the genetic determinants of lung cancer, Wang et al. performed a meta-analysis involving 1018 publications that analyzed 2910 genetic variants located in 754 different genes or chromosome loci. Here, 22 variants of the 21 genes (*APEX1* rs1130409 and rs1760944, *ATM* rs664677, *AXIN2* rs2240308, *CHRNA3* rs6495309, *CHRNA5* rs16969968, *CLPTM1L* rs402710, *CXCR2* rs1126579, *CYP1A1* rs4646903, *CYP2E1* rs6413432, *ERCC1* rs11615, *ERCC2* rs13181, *FGFR4* rs351855, *HYKK* rs931794, *MIR146A* rs2910164, *MIR196A2* rs11614913, *OGG1* rs1052133, *PON1* rs662, *REV3L* rs462779, *SOD2* rs4880, *TERT* rs2736098, and *TP53* rs1042522) were found to be significantly associated with lung cancer susceptibility [40].

To the best of our knowledge, our study is the first to observe the importance of the *CASP-8* polymorphism as a genetic factor predisposing to the development of NSCLC in smokers and in those with a family history of cancer. We also observed that the GG genotype rs3769818 is significantly more frequent in patients with advanced disease and with distant metastases (*p* = 0.0221). The study by Liao et al. analyzed the effect of caspase 8 expression on the risk of distant metastases in a group of 203 NSCLC patients. Brain metastases were identified in 16.1% (18/112) of patients in the high-caspase-8-expression group and only 1.1% (1/91) in the low-expression group. There was also a correlation between lymph node metastases and caspase 8 levels (*p* = 0.08). Increased caspase 8 levels predicted early metastases to the brain (log-rank test, *p* = 0.00) [41].

Our observations produce further evidence that caspase 8 may be involved in the progression and metastasis process of NSCLC. We also observed an effect of the studied rs3769818 *CASP-8* variant on the treatment outcomes in patients with NSCLC who underwent chemotherapy with platinum derivatives. We showed that the presence of the rs3769818 GG variant is associated with a significantly higher risk of disease progression. In carriers of the GG genotype of the *CASP-8* gene, we observed a higher risk of PFS and TTP shortening. The influence of the GG genotype of the *CASP-8* gene on the PFS was confirmed in a multivariate analysis. Thus, patients with this specific genotype should perhaps receive other treatment regimens or require additional clinical support because their therapy may be less effective. However, we did not confirm the influence of the tested factors on the OS.

In the literature, there are single reports presenting SNPs of the *CASP-8* gene as prognostic factors in lung cancer patients. In fact, the available data (from breast tissue) indicate that it is the AA and not the GG genotype of the *CASP-8* gene that is usually associated with lower (and therefore generally unfavorable, apoptosis-inhibiting) expression of the encoded protein [42]. However, it should be taken into account that this gene may be regulated differently in different tissues and different types of cancer. This is confirmed by [43] showing differences in this respect in SCLC and NSCLC. In NSCLC, we usually observe high, not low, expression of this gene and protein, although it is low expression that should be associated with the development of this tumor, a higher risk of metastasis, and disease progression [14,22]. In this context, the conclusion drawn from the work of Terlizzi et al. [14] that pharmacological inhibition of caspase 8 reduces tumor growth by reducing the release of pro-inflammatory cytokines seems especially interesting because it suggests that the initial genotype that conditions high expression may lead to worse prognosis in NSCLC.

Liu et al. proved that the SNP variants rs3769821 and rs1045494 of *CASP-8* have a significant impact on the overall survival in patients with lung adenocarcinoma. In patients with the AA rs3769821 genotype, the OS was 6.7 months longer than in carriers of the GG or AG genotype (*p* = 0.007). The authors analyzed the effect of the haplotype of seven *CASP-8* tapSNPs on the OS. A relationship was observed between the AGGAAAGA haplotype and the overall survival in patients with pulmonary adenocarcinoma. Patients with no copy had an OS 6 months longer than patients with one or two copies of the AGGAAAGA haplotype (zero copies, median OS = 23.9 months; 1–2 copies, median OS = 17.7 months; *p* = 0.016). There was no correlation between the *CASP-8* polymorphisms/haplotypes and the PFS [44].

## 5. Conclusions

Our findings suggest that the rs3769818 *CASP-8* polymorphic variant may be a genetic factor predisposing to the development of lung cancer. It may act as a biomarker to identify patients at high risk of metastatic disease and a predictive factor in NSCLC patients receiving platinum-based chemotherapy. It is justified to undertake further research on a larger population of patients in this new, extremely interesting research direction.

## Figures and Tables

**Table 1:** Characteristics of the study group.

Factor		Study Group (*n* = 99)
Gender	Male	76 (76.77%)
	Female	23 (23.23%)
Age, median (range)		66.5 (44–83)
	≥66.5	49 (49.50%)
	<66.5	50 (50.50%)
Disease stage	III	35(35.35%)
	IV	64 (64.65%)
Distant metastases	No	43 (43.43%)
	Yes	56 (56.57%)
Histopathology	AC	40 (40.40%)
	SCC	52 (52.53%)
	NOS	7 (7.07%)
Performance status (ECOG score)	0	17(17.17%)
	1	58 (58.59%)
	2	24 (24.24%)
Smoking status	Smoker	92 (92.93%)
	Non-smoker	7 (7.07%)
Pack-years, median (range)		45.00 (1–100)
	≥45	45 (45.45%)
	<45	54 (54.55%)
Weight, median (range)		74 (46–117)
	≥74	49 (49.49%)
	<74	50 (50.51%)
BMI, median (range)		24.91 (15.02–40.88)
	≥24.91	49 (49.49%)
	<24.91	50 (50.51%)
Body loss (%), median (range)		9.84(0–40)
	≥9.84	32 (32.32%)
	<9.84	67 (67.68%)
Time from diagnosis to treatment (days)		17.00 (3–217)
	≥17	48 (48.48%)
	<17	51 (51.52%)
First-line chemotherapy (I)	Cis + PEM	23 (23.23%)
	PN	60 (60.60%)
	PG	16 (16.16%)
Number of CTH cycles	1–3	38 (38.38%)
	4–8	61 (61.62%)
First evaluation after first-line chemotherapy	PR	36 (48.65%)
	SD	29 (39.19%)
	PD	9 (12.16%)
Second evaluation after first-line chemotherapy	PR	10 (20.00%)
	SD	32 (64.00%)
	PD	8 (16.00%)
Occupational exposure	No	55 (79.71%)
	Yes	14 (20.29%)
Family history of malignancy (any)	No	46 (46.46%)
	Yes	53 (53.54%)
Family history of malignancy (lung)	No	75 (75.76%)
	Yes	24 (24.24%)

Abbreviations: AC—adenocarcinoma; Cis—cisplatin; CTH—chemotherapy; NOS—not otherwise specified; SCC—squamous cell carcinoma; BMI—body mass index; ECOG—Eastern Cooperative Oncology Group; PD—progressive disease; PEM—pemetrexed; PG—cisplatin + gemcitabine; PN—cisplatin + vinorelbine; PR—partial response; SD—stable disease.

**Table 2:** Distribution of *CASP-8* genotypes according to demographic and clinical factors.

Variable		*CASP-8* (rs3769818)			
		AA (*n* = 8)	AG (*n* = 38)	GG (*n* = 53)	*p*-Value χ^2^
Gender	Male	5 (6.58%)	32 (42.11%)	39 (51.31%)	0.3020 2.39
	Female	3 (13.04%)	6 (26.09%)	14 (60.87%)	
Age	≥66.5	4 (81.16%)	21 (42.86%)	24 (48.98%)	0.6432 0.88
	<66.5	4 (8.00%)	17 (34.00%)	29 (58.00%)	
Disease stage	III	3 (8.58%)	16 (45.71%)	16 (45.71%)	0.4984 1.39
	IV	5 (7.81%)	22 (34.38%)	37 (57.81%)	
Distant metastases	No	1 (2.56%)	21 (53.85%)	17 (43.59%)	0.0221 7.62 *
	Yes	7 (11.67%)	17 (28.33%)	36 (60.00%)	
Histopathology	AC	5 (12.50%)	18 (45.00%)	17 (42.50%)	0.2165 5.78
	SCC	3 (5.77%)	16 (30.78%)	33 (63.46%)	
	NOS	-	4 (57.14%)	3 (42.86%)	
Performance status (ECOG score)	0	1 (5.88%)	11 (64.71%)	5 (29.41%)	0.1178 7.36
	1	4 (6.90%)	18 (31.03%)	36 (62.07%)	
	2	3 (12.50%)	9 (37.50%)	12 (50.00%)	
Smoking status	Smoker	5 (5.43%)	37 (40.22%)	52 (56.52%)	<0.0001 19.14 *
	Non-smoker	3 (42.86%)	1 (28.57%)	1 (28.57%)	
Body loss (%)	≥9.84	1 (3.13%)	9 (28.12%)	22 (68.75%)	0.0917 4.78
	<9.84	7 (10.45%)	29 (43.28%)	31 (46.27%)	
Family history of malignancy (any)	No	-	27 (57.45%)	20 (42.55%)	0.0003 16.00 *
	Yes	8 (15.69%)	12 (23.53%)	31 (60.78%)	
Family history of malignancy (lung)	No	3 (6.38%)	25 (53.19%)	19 (40.46%)	0.0157 8.30 *
	Yes	5 (9.62%)	13 (25.00%)	34 (65.38%)	

Abbreviations: AC—adenocarcinoma; NOS—not otherwise specified; SCC—squamous cell carcinoma. * Statistically significant results.

**Table 3:** Risk of disease progression according to demographic, clinical, and genetic factors (first evaluation).

Variable		Objective Response #		OR (95% CI)	*p*-Value
		PD (*n* = 9)	PR and SD (*n* = 65)		
Gender	Male	6 (10.00%)	54 (90.00%)	0.41 (0.091.88)	0.2500
	Female	3 (21.43%)	11 (78.57%)		
Age	≥66.5	3 (9.37%)	29 (90.63%)	1.24 (0.23–6.62)	0.8001
	<66.5	6 (14.28%)	36 (85.72%)		
Disease stage	III	1 (3.57%)	27 (96.43%)	5.68 (0.67–48.15)	0.1109
	IVA	8 (17.39%)	38 (82.61%)		
Performance status (ECOG score)	0	2 (18.18%)	9 (81.82%)	1.78 (0.32–9.95)	0.5125
	1 and 2	7 (11.11%)	56 (88.89%)		
Smoking status	Smoker	8 (11.59%)	61 (88.41%)	0.52 (0.05–5.29)	0.5844
	Non-smoker	1 (20.00%)	4 (80.00%)		
Pack-years	≥45	4 (11.11%)	32 (88.89%)	0.82 (0.20–3.35)	0.7880
	<45	5 (13.16%)	33 (86.84%)		
First-line chemotherapy (I)	Cis + PEM	3 (17.65%)	14 (82.35%)	1.82 (0.40–8.22)	0.4354
	Other	6 (10.53%)	51 (89.47%)		
First-line chemotherapy (II)	PN	5 (10.42%)	43 (89.58%)	0.64 (0.15–2.62)	0.5348
	Other	4 (15.38%)	22 (84.62%)		
First-line chemotherapy (III)	PG	1 (14.29%)	6 (85.71%)	1.23 (0.13–11.57)	0.8028
	Other	8 (11.94%)	59 (88.06%)		
Distant metastases	No	1 (4.35%)	22 (95.65%)	0.24 (0.03–2.08)	0.1971
	Yes	8 (15.69%)	43 (84.31%)		
Weight	≥74	1 (2.94%)	33 (97.06%)	0.12 (0.01–1.02)	0.0527
	<74	8 (20.00%)	32 (80.00%)		
BMI	≥24.91	1 (3.03%)	32 (96.97%)	0.13 (0.01–1.09)	0.0600
	<24.91	8 (19.51%)	33 (80.49%)		
Body loss (%)	≥9.84	6 (24.00%)	19 (76.00%)	4.84 (1.09–21.39)	0.0374 *
	<9.84	3 (6.52%)	46 (93.48%)		
Time from diagnosis to treatment (days)	≥17	5 (14.28%)	30 (85.72%)	1.4583 (0.36–5.93)	0.5980
	<17	4 (10.27%)	35 (89.73%)		
Occupational exposure	No	7 (12.73%)	48 (87.27%)	1.90 (0.21–16.82)	0.5658
	Yes	1 (7.14%)	13 (92.86%)		
Family history of malignancy (any)	No	2 (5.56%)	34 (94.44%)	0.26 (0.05–1.35)	0.1090
	Yes	7 (18.42%)	31 (81.58%)		
Family history of malignancy (lung)	No	6 (11.11%)	48 (88.89%)	0.71 (0.16–3.15)	0.6506
	Yes	3 (15.00%)	17 (85.00%)		
Histopathology (I)	AC	4 (13.79%)	25 (86.21%)	1.28 (0.31–5.22)	0.7309
	SCC and NOS	5 (11.11%)	40 (88.89%)		
Histopathology (II)	SCC	5 (12.50%)	35 (87.50%)	1.07 (0.26–4.35)	0.9232
	AC and NOS	4 (11.76%)	30 (88.24%)		
*CASP-8* (rs3769818)	AA	1 (16.67%)	6 (83.33%)	1.23 (0.13–11.57)	0.8569
	AG and GG	8 (11.94%)	59 (88.06%)		
*CASP-8* (rs3769818)	AG	-	28 (100.00%)	0.07 (0.01–1.24)	0.0697
	AA and GG	9 (19.57%)	37 (80.43%)		
*CASP-8* (rs3769818)	GG	8 (20.51%)	31 (79.49%)	8.77 (1.04–74.21)	0.0462 *
	AG and AA	1 (2.86%)	34 (97.14%)		

Abbreviations: AC—adenocarcinoma; BMI—body mass index; Cis—cisplatin; CTH—chemotherapy; NOS—not otherwise specified; OR—odds ratio; SCC—squamous cell carcinoma; PD—progressive disease; PEM—pemetrexed; PG—cisplatin + gemcitabine; PN—cisplatin + vinorelbine; PR—partial response; SD—stable disease. * Statistically significant results. ^#^ In some cases (*n* = 25), reliable determination of the response in the first evaluation (after the second CTH cycle) was not possible (too short follow-up—discontinuation of treatment prior to assessment due to poor tolerance or lack of appearance at the scheduled assessment appointment). However, there were no statistically significant differences in the data distribution when the entire study group (*n* = 99) and a group of patients included in the first assessment (*n* = 74) were compared (Table S1).

**Table 4:** Risk of disease progression according to demographic, clinical, and genetic factors (second evaluation).

Variable		PD (*n* = 8)	PR and SD (*n* = 42)	OR (95% CI)	*p*-Value
Gender	Male	5(12.20%)	37 (87.80%)	0.22 (0.04–1.24)	0.0874
	Female	3(37.50%)	5(62.50%)		
Age	≥66.5	4(21.05%)	15 (78.95%)	1.80 (0.39–8.25)	0.4494
	<66.5	4(12.90%)	27 (87.10%)		
Disease stage	III	1(4.76%)	20 (95.24%)	0.16 (0.02–1.39)	0.0963
	IV	7 (24.18%)	22 (78.86%)		
Performance status (ECOG score)	0	0(0.00%)	9(100.00%)	0.21 (0.01–3.93)	0.2946
	1 and 2	8 (19.51%)	33 (80.49%)		
Smoking status	Smoker	8 (17.02%)	39(82.98%)	1.59 (0.07–31.95	0.7926
	Non-smoker	0(0.00%)	3(100.00%)		
Pack-years	≥45	4(17.39%)	19(82.61%)	1.21 (0.27–5.50)	0.8046
	<45	4(14.81%)	23(85.19%)		
First-line chemotherapy (I)	Cis + PEM	3(23.08%)	10(76.92%)	1.26 (0.25–6.35)	0.7794
	Other	5(19.23%)	21(80.77%)		
First-line chemotherapy (II)	PN	4(12.50%)	28(87.50%)	0.50 (0.11–2.30)	0.3737
	Other	4 (22.22%)	14(77.78%)		
First-line chemotherapy (III)	PG	1(33.33%)	2(66.67%)	2.86 (0.23–35.91)	0.4163
	Other	7 (14.89%)	40 (85.11%)		
Number of cycles	1–3	2 (28.57%)	5 (71.43%)	2.46 (0.39–15.73)	0.3395
	4–8	6 (13.95%)	37(86.05%)		
Metastases	No	1(5.88%)	16 (94.11%)	0.23 (0.03–2.07)	0.1904
	Yes	7 (21.21%)	26 (78.79%)		
Weight	≥74	5 (18.52%)	22 (81.48%)	1.51 (0.32–7.17)	0.6003
	<74	3 (13.04%)	20 (86.96%)		
BMI	≥24.91	6 (22.22%)	21 (77.78%)	3.00 (0.54–16.60)	0.2082
	<24.91	2(8.70%)	21 (91.30%)		
Body weight loss (%)	≥9.84	7(46.67%)	8(53.33%)	29.75 (3.19–277.33)	0.0029 *
	<9.84	1(2.86%)	34(97.14%)		
Time from diagnosis to treatment (days)	≥17	7 (28.00%)	18 (72.00%)	9.33 (1.05–82.78)	0.0449 *
	<17	1(4.00%)	24 (96.00%)		
Occupational exposure	No	6 (15.79%)	32 (84.21%)	0.94 (0.16–5.40)	0.9424
	Yes	2 (16.67%)	10 (83.33%)		
Family history of malignancy (any)	No	4 (17.39%)	19 (82.61%)	1.21 (0.27–5.50)	0.8046
	Yes	4 (10.81%)	23 (89.19%)		
Family history of malignancy (lung)	No	1(7.14%)	13 (92.86%)	0.26 (0.02–2.85)	0.2681
	Yes	3 (23.08%)	10 (76.92%)		
Histopathology (I)	AC	4 (22.22%)	14 (77.78%)	2.00 (0.43–9.21)	0.3737
	SCC and NOS	4 (12.50%)	28 (87.50%)		
Histopathology (II)	SCC	4 (14.81%)	23 (85.19%)	0.83 (0.18–3.75)	0.8046
	AC and NOS	4 (17.39%)	19 (82.61%)		
*CASP-8* (rs3769818)	AA	0(0.00%)	3 (100.00%)	0.66 (0.03–14.08)	0.7926
	AG and GG	8 (17.02%)	39 (82.98%)		
*CASP-8* (rs3769818)	AG	1(5.26%)	18 (94.74%)	0.19 (0.02–1.69)	0.1365
	AA and GG	7 (22.58%)	24 (77.42%)		
*CASP-8* (rs3769818)	GG	7 (29.17%)	17(70.83%)	10.29 (1.16–91.43)	0.0364 *
	AG and AA	1(3.85%)	25(96.15%)		

Abbreviations: AC—adenocarcinoma; BMI—body mass index; Cis—cisplatin; CTH—chemotherapy; NOS—not otherwise specified; OR—odds ratio; SCC—squamous cell carcinoma; PD—progressive disease; PEM—pemetrexed; PG—cisplatin + gemcitabine; PN—cisplatin + vinorelbine; PR—partial response; SD—stable disease. * Statistically significant results. ^#^ In some cases (*n* = 49), reliable determination of the response in the second evaluation (after the second CTH cycle) was not possible (too short follow-up—discontinuation of treatment prior to assessment due to poor tolerance or lack of appearance at the scheduled assessment appointment). However, there were no statistically significant differences in the data distribution when the entire study group (*n* = 99) and a group of patients included in the second assessment (*n* = 50) were compared (Table S1).

**Table 5:** Progression-free survival, time to progression, and overall survival according to demographic, clinical, and genetic factors.

Variable		Survival Analysis ^#^ (*n* = 89)					
		Progression-Free Survival		Time to Progression		Overall Survival	
		Median (Days)	*p*-Value HR (95% CI)	Median (Days)	*p*-Value HR (95% CI)	Median (Days)	*p*-Value HR (95% CI)
Gender	Male Female	41.00 131.00	0.3903 1.26 (0.71–2.23) 0.80 (0.45–1.41)	54.00 164.00	0.2553 1.34 (0.77–2.34) 0.75 (0.43–1.30)	282.00 332.00	0.9624 1.01 (0.59–1.73) 0.99 (0.58–1.69)
Age	≥66.5 <66.5	84.00 122.00	0.1941 1.33 (0.85–2.07) 0.75(0.48–1.17)	117.00 167.00	0.2033 1.31 (0.86–2.02) 0.76 (0.49–1.17)	249.00 389.00	0.1539 1.37 (0.88–2.11) 0.73 (0.47–1.13)
Disease stage	III IV	198.00 68.00	<0.0001 * 0.40 (0.26–0.62) 2.50 (1.61–3.89)	222.00 92.00	<0.0001 * 0.39 (0.25–0.60) 2.56 (1.67–3.92)	502.00 228.00	0.0021 * 0.50 (0.32–0.77) 2.01 (1.30–3.09)
Performance status (ECOG score)	0 1 and 2	182.00 95.00	0.4665 0.74 (0.35–1.54) 1.35 (0.65–2.82)	190.00 134.00	0.5332 0.77 (0.36–1.62) 1.30 (0.62–2.74)	389.00 233.00	0.1058 0.51 (0.27–0.97) 1.96 (1.03–3.71)
Smoking status	Smoker Non-smoker	36.00 107.00	0.6478 1.23(0.46–3.30) 0.81 (0.30–2.19)	51.00 145.00	0.73201.17 (0.44–3.08) 0.86 (0.32–2.25)	321.00 412.00	0.3434 1.54 (0.72–3.27) 0.65 (0.31–1.38)
Pack-years	<45 ≥45	122.00 89.00	0.0513 0.67 (0.43–1.03) 1.50 (0.97–2.30)	167.00 114.00	0.0408 * 0.65 (0.42–1.00) 1.54 (1.00–2.36)	337.00 321.00	0.3978 0.83 (0.54–1.28) 1.20 (0.78–1.86)
First-line chemotherapy (I)	Cis + Pem Other	61.00 122.00	0.0356 * 1.64 (0.94–2.88) 0.61 (0.35–1.06)	70.00 149.00	0.0240 * 1.72(0.97–3.03) 0.58 (0.33–1.03)	233.00 337.00	0.0638 1.58 (0.90–2.78) 0.63 (0.36–1.11)
First-line chemotherapy (II)	PN Other	122.00 76.00	0.,0198 * 0.61 (0.38–0.97) 1.64 (1.03–2.62)	167.00 115.00	0.0380 * 0.64 (0.40–1.02) 1.56 (0.98–2.49)	332.00 237.00	0.6685 0.91 (0.58–1.42) 1.10 (0.70–1.72)
First-line chemotherapy (III)	PG Other	68.00 122.00	0.3377 1.35 (0.66–2.76) 0.74 (0.36–1.51)	115.00 162.00	0.7066 1.13 (0.58–2.20) 0.89 (0.45–1.72)	355.00 337.00	0.9503 0.98 (0.50–1.89) 1.02 (0.53–1.98)
Number of cycles	1–3 4–8	40.00 213.00	<0.0001 * 4.24 (2.07–8.72) 0.23 (0.11–0.48)	58.00 237.00	<0.0001 * 3.80 (1.90–7.59) 0.26 (0.13–0.53)	137.00 472.00	0.0005 * 2.39 (1.31–4.36) 0.42 (0.23–0.76)
Metastases	No Yes	213.00 61.00	0.0016 * 0.43 (0.27–0.70) 2.30 (1.42–3.72)	237.00 78.00	0.0013 * 0. 42 (0.26–0.69) 2.36 (1.46–3.82)	502.00 224.00	0.0026 * 0.44 (0.27–0.72) 2.28 (1.40–3.73)
Weight	≥74 <74	122.00 89.00	0.7208 0.93 (0.60–1.42) 1.08 (0.70–1.65)	145.00 139.00	0.7114 0.92 (0.60–1.41) 1.08 (0.71–1.66)	337.00 274.00	0.4882 0.86 (0.56–1.32) 1.16 (0.75–1.80)
BMI	≥24.91 <24.91	122.00 82.00	0.9569 1.01(0.66–1.55) 0.99 (0.64–1.51)	131.00 151.00	0.8788 1.03 (0.68–1.58) 0.97 (0.63–1.48)	337.00 250.00	0.7648 0.94 (0.61–1.44) 1.07 (0.69–1.65)
Body weight loss (%)	<9.84 ≥9.84	122.00 47.00	0.2319 0.76 (0.47–1.23) 1.31 (0.81–2.12)	167.00 78.00	0.2424 0.76 (0.47–1.24) 1.31 (0.81–2.12)	438.00 168.00	0.0428 * 0.62 (0.37–1.05) 1.60 (0.95–2.69)
Time from diagnosis to treatment (days)	≥17 <17	92.00 122.00	0.0504 1.50 (0.97–2.31) 0.67 (0.43–1.03)	149.00 134.00	0.2929 1.25 (0.81–1.91) 0.80 (0.52–1.23)	337.00 282.00	0.6745 0.91 (0.59–1.41) 1.10 (0.71–1.69)
Occupational exposure	No Yes	95.00 105.00	0.5610 1.20 (0.66–2.18) 0.83 (0.46–1.52)	139.00 164.00	0.6304 1.16 (0.64–2.13) 0.86 (0.47–1.57)	282.00 350.00	0.8392 1.07 (0.57–1.99) 0.94 (0.50–1.74)
Family history of malignancy (any)	No Yes	137.00 91.00	0.5386 0.88 (0.57–1.35) 1.14 (0.74–1.75)	177.00 132.00	0.5338 0.87 (0.57–1.34) 1.14 (0.75–1.75)	273.00 321.00	0.7529 1.07 (0.69–1.66) 0.93 (0.60–1.44)
Family history of malignancy (lung)	No Yes	107.00 83.00	0.7111 0.89 (0.47–1.67) 1.12 (0.60–2.10)	135.00 115.00	0.7796 0.92 (0.49–1.71) 1.09 (0.58–2.05)	438.00 168.00	0.3720 0.75 (0.40–1.42) 1.33 (0.70–2.52)
Histopathology (I)	SCC AC and NOS	84.00 137.00	0.7849 1.07 (0.64–1.81) 0.93 (0.55–1.57)	117.00 167.00	0.9363 1.02 (0.60–1.73) 0.98 (0.58–1.65)	355.00 337.00	0.9455 1.02 (0.58–1.77) 0.98 (0.56–1.71)
Histopathology (II)	AC SCC and NOS	137.00 84.00	0.4433 0.82 (0.49–1.37) 1.22 (0.73–2.04)	167.00 117.00	0.5556 0.85 (0.51–1.43) 1.17 (0.70–1.96)	337.00 355.00	0.9228 1.03 (0.59–1.79) 0.97 (0.56–1.69)
*CASP-8* (rs3769818)	AA AG and GG	365.00 95.00	0.0035 * 0.36 (0.21–0.61) 2.78 (1.65–4.76)	135.00 458.00	0.0029 * 0.35 (0.20–0.59) 2.88(1.70–4.88)	631.00 273.00	0.1757 0.61 (0.33–1.12) 1.64 (0.89–3.00)
*CASP-8* (rs3769818)	AG AA and GG	152.00 84.00	0.0105 * 0.56 (0.36–0.86) 1.79 (1.16–2.75)	222.00 114.00	0.0117 * 0.56 (0.36–0.86) 1.79 (1.17–2.76)	332.00 321.00	0.3665 1.26 (0.76–2.08) 0.79 (0.48–1.31)
*CASP-8* (rs3769818)	GG AG and AA	82.00 198.00	0.0152 * 1.68 (1.10–2.57) 0.59 (0.39–0.91)	108.00 222.00	0.0151 * 1.70 (1.11–2.60) 0.59 (0.38–0.91)	237.00 502.00	0.0601 1.52 (0.98–2.34) 0.66 (0.43–1.02)

Abbreviations: AC—adenocarcinoma; BMI—body mass index; Cis—cisplatin; SCC—squamous cell carcinoma; PEM—pemetrexed; PG—cisplatin + gemcitabine; PN—cisplatin + vinorelbine; HR, hazard ratio. * Statistically significant results. ^#^ In some cases (*n* = 10), reliable determination of the survival time was not possible (too short follow-up—discontinuation of treatment prior to assessment time due to poor tolerance, failure to appear at the next appointment, or any contact with a patient lost). However, there were no statistically significant differences in the data distribution when the entire study group (*n* = 99) and a group of patients included in survival analysis (*n* = 89) were compared (Table S1).

**Table 6:** Cox’s logistic regression analysis for progression-free survival, time to progression, and overall survival.

Variable		Survival Analysis ^#^ (*n* = 89)		
		Progression-Free Survival	Time to Progression	Overall Survival
		*p*-Value HR (95% CI)	*p*-Value HR (95% CI)	*p*-Value HR (95% CI)
Gender	Female	0.0689 0.47 (0.21–1.06)	0.9032 0.95 (0.42–2.14)	0.9976 0.99 (0.50–2.00)
Age	<66.5	0.1160 0.64 (0.37–1.11)	0.0952 0.61 (0.34–1.09)	0.1312 0.66 (0.38–1.13)
Disease stage	IV	0.0016 * 2.56 (1.43–7.76)	0.0028 * 2.44 (1.35–4.35)	0.0002 * 2.94 (1.64–5.26)
Performance status (ECOG score)	1 and 2	0.5521 0.71 (0.23–2.19)	0.6948 0.79 (0.24–2.57)	0.0917 2.54 (0.86–7.49)
Smoking status	Smoker	0.3681 0.63 (0.23–1.71)	0.9254 1.05 (0.39–2.79)	0.3583 1.56 (0.60–4.01)
Pack-years	≥45 (years)	0.4021 1.24 (0.75–2.05)	0.3709 1.25 (0.77–2.04)	0.7364 0.92 (0.55–1.53)
First-line chemotherapy (I)	Cis + PEM	0.8361 0.92 (0.41–2.04)	0.3604 0.69 (0.31–1.54)	0.4484 0.80 (0.45–1.42)
First-line chemotherapy (II)	PN	0.4374 1.22 (0.74–2.00)	0.6315 1.13 (0.68–1.87)	0.1687 0.69 (0.41–1.17)
First-line chemotherapy (III)	PG	0.8369 1.09 (0.49–2.42)	0.3636 1.46 (0.65–3.27)	0.1728 1.69 (0.79–3.57)
Number of chemotherapy cycles	4–8	0.5711 0.74 (0.26–2.08)	0.4788 0.68 (0.24–1.97)	0.7475 1.19 (0.41–3.41)
Distant metastases	Yes	0.0353 * 3.16 (1.09–9.15)	0.0514 2.95 (0.99–8.75)	0.1656 2.09 (0.74–5.91)
Weight	≥74 (kg)	0.1160 0.64 (0.37–1.11)	0.4989 1.19 (0.72–1.96)	0.1433 1.49 (0.87–2.54)
BMI	<24.91	0.4987 0.99 (0.94–1.03)	0.4333 1.23 (0.73–2.05)	0.0474 * 1.72 (1.01–2.95)
Body loss (%)	≥9.84 (%)	0.5747 1.16 (0.69–1.94)	0.1384 1.56 (0.87–2.81)	0.0202 * 1.95 (1.11–3.44)
Time from diagnosis to treatment (days)	≥17 (days)	0.7208 0.91 (0.55–1.51)	0.3378 1.29 (0.76–2.19)	0.3286 1.32 (0.76–2.28)
Occupational exposure	Yes	0.5177 1.31 (0.58–2.93)	0.2608 1.61 (0.70–3.72)	0.5984 1.25 (0.55–2.86)
Family history of malignancy (any)	Yes	0.4368 1.14 (0.74–1.75)	0.1450 1.49 (0.87–2.53)	0.4585 0.81 (0.46–1.42)
Family history of malignancy (lung)	Yes	0.8379 1.11 (0.40–3.10)	0.5220 0.75 (0.32–6.75)	0.5430 0.76 (0.31–1.85)
Histopathology	SCC	0.2543 1.81 (0.65–5.02)	0.0167 * 3.87 (1.28–11.70)	0.1252 1.73 (0.86–3.50)
*CASP-8* (rs3769818)	AA	0.1084 0.44 (0.16–1.19)	0.0506 0.36 (0.13–1.00)	0.8303 1.11 (0.42–2.90)
*CASP-8* (rs3769818)	AG	0.2769 0.69 (0.35–1.35)	0.7188 0.89 (0.46–1.72)	0.4396 0.78 (0.42–1.46)
*CASP-8* (rs3769818)	GG	0.0317 * 1.80 (1.06–3.05)	0.5372 1.19 (0.69–2.06)	0.9805 1.01 (0.58–1.75)

Abbreviations: BMI—body mass index; Cis—cisplatin; CTH—chemotherapy; SCC—squamous cell carcinoma; PEM—pemetrexed; PG—cisplatin + gemcitabine; PN—cisplatin + vinorelbine. * Statistically significant results. ^#^ In some cases (*n* = 10), reliable determination of the survival time was not possible (too short follow-up—discontinuation of treatment prior to assessment time due to poor tolerance, failure to appear at the next appointment, or any contact with a patient lost). However, there were no statistically significant differences in the data distribution when the entire study group (*n* = 99) and a group of patients included in survival analysis (*n* = 89) were compared (Table S1).

## Data Availability

Not applicable.

## Supplementary Materials

The following are available online at https://www.mdpi.com/2077-0383/10/5/1126/s1, Table S1: Comparisons of data distribution according to different study group sets.

## References

[1] Fennell D.A. (2005). Caspase Regulation in Non–Small Cell Lung Cancer and its Potential for Therapeutic Exploitation. Clin. Cancer Res..

[2] Ferreira C.G., Span S.W., Peters G.J., Kruyt F.A., Giaccone G. (2000). Chemotherapy triggers apoptosis in a caspase-8-dependent and mitochon-dria-controlled manner in the non-small cell lung cancer cell line NCI-H460. Cancer Res..

[3] Paul I., Chacko A.D., Stasik I., Busacca S., Crawford N., McCoy F., McTavish N., Wilson B., Barr M., O’Byrne K.J. (2012). Acquired differential regulation of caspase-8 in cisplatin-resistant non-small-cell lung cancer. Cell Death Dis..

[4] Panov S.Z. (2005). Molecular biology of the lung cancer. Radiol. Oncol..

[5] Massion P.P., Carbone D.P. (2003). The molecular basis of lung cancer: Molecular abnormalities and therapeutic implications. Respir. Res..

[6] Hanahan D., Weinberg R.A. (2000). The hallmarks of cancer. Cell.

[7] McIlwain D.R., Berger T., Mak T.W. (2013). Caspase functions in cell death and disease. Cold Spring Harb. Perspec. Biol..

[8] Chang D.W., Xing Z., Capacio V.L., Peter M.E., Yang X. (2003). Interdimer processing mechanism of procaspase-8 activation. EMBO J..

[9] Wilson N.S., Dixit V., Ashkenazi A. (2009). Death receptor signal transducers: Nodes of coordination in immune signaling networks. Nat. Immunol..

[10] Gurung P., Anand P.K., Malireddi R.K.S., Walle L.V., Van Opdenbosch N., Dillon C.P., Weinlich R., Green D.R., Lamkanfi M., Kanneganti T.-D. (2014). FADD and Caspase-8 Mediate Priming and Activation of the Canonical and Noncanonical Nlrp3 Inflammasomes. J. Immunol..

[11] Kaiser W.J., Upton J.W., Long A.B., Livingston-Rosanoff D., Daley-Bauer L.P., Hakem R., Caspary T., Mocarski E.S. (2011). RIP3 mediates the embryonic lethality of caspase-8-deficient mice. Nat. Cell Biol..

[12] Oberst A., Dillon C.P., Weinlich R., McCormick L.L., Fitzgerald P., Pop C., Hakem R., Salvesen G.S., Green D.R. (2011). Catalytic activity of the caspase-8-FLIP(L) complex inhibits RIPK3-dependent ne-crosis. Nature.

[13] Kawadler H., Gantz M.A., Riley J.L., Yang X. (2008). The Paracaspase MALT1 Controls Caspase-8 Activation during Lymphocyte Proliferation. Mol. Cell.

[14] Terlizzi M., Di Crescenzo V.G., Perillo G., Galderisi A., Pinto A., Sorrentino R. (2015). Pharmacological inhibition of caspase-8 limits lung tumour outgrowth. Br. J. Pharmacol..

[15] Herrera L.A., Benítez-Bribiesca L., Mohar A., Ostrosky-Wegman P. (2005). Role of infectious diseases in human carcinogenesis. Environ. Mol. Mutagen..

[16] Soung Y.H., Lee J.W., Kim S.Y., Jang J., Park Y.G., Park W.S., Nam S.W., Lee J.Y., Yoo N.J., Lee S.H. (2005). CASPASE-8 gene is inactivated by somatic mutations in gastric carcinomas. Cancer Res..

[17] Fulda S. (2009). Caspase-8 in cancer biology and therapy. Cancer Lett..

[18] Kim H.S., Lee J.W., Soung Y.H., Park W.S., Kim S.Y., Lee J.H., Park J.Y., Cho Y.G., Kim C.J., Jeong S.W. (2003). Inactivating mutations of caspase-8 gene in colorectal carcinomas. Gastroenterology.

[19] Duiker E.W., Meijer A., Van Der Bilt A.R.M., Meersma G.J., Kooi N., Van Der Zee A.G.J., De Vries E.G., De Jong S. (2011). Drug-induced caspase 8 upregulation sensitises cisplatin-resistant ovarian carcinoma cells to rhTRAIL-induced apoptosis. Br. J. Cancer.

[20] Van Geelen C.M., Pennarun B., Ek W.B., Le P.T.K., Spierings D.C.J., De Vries E.G.E., De Jong S. (2010). Downregulation of active caspase 8 as a mechanism of acquired TRAIL re-sistance in mismatch repair-proficient colon carcinoma cell lines. Int. J. Oncol..

[21] Shivapurkar N., Reddy J., Matta H., Sathyanarayana U.G., Huang C.X., Toyooka S., Minna J.D., Chaudhary P.M., Gazdar A.F. (2002). Loss of expression of death-inducing signaling complex (DISC) components in lung cancer cell lines and the influence of MYC amplification. Oncogene.

[22] Riley J.S., Hutchinson R.W., McArt D.G., Crawford N.P.S., Holohan C., Paul A.I., Van Schaeybroeck S., Saltotellez M., Johnston P.G., Fennell A.D. (2013). Prognostic and therapeutic relevance of FLIP and procaspase-8 overexpression in non-small cell lung cancer. Cell Death Dis..

[23] Grenet J., Teitz T., Wei T., Valentine V., Kidd V.J. (1999). Structure and chromosome localization of the human CASP8 gene. Gene.

[24] Son J.-W., Kang H.-K., Chae M.H., Choi J.E., Park J.M., Lee W.K., Kim C.H., Kim D.S., Kam S., Kang Y.M. (2006). Polymorphisms in the caspase-8 gene and the risk of lung cancer. Cancer Genet. Cytogenet..

[25] Hart K., Landvik N.E., Lind H., Skaug V., Haugen A., Zienolddiny S. (2011). A combination of functional polymorphisms in the CASP8, MMP1, IL10 and SEPS1 genes affects risk of non-small cell lung cancer. Lung Cancer.

[26] Ulybina Y.M., Kuligina E.S., Mitiushkina N.V., Rozanov M.E., Ivantsov A.O., Ponomariova D.N., Togo A.V., Levchenko E.V., Shutkin V.A., Brenister S.I. (2009). Coding polymorphisms in Casp5, Casp8 and DR4 genes may play a role in predisposition to lung cancer. Cancer Lett..

[27] Zhang R., Chu M., Zhao Y., Wu C., Guo H., Shi Y., Dai J., Wei Y., Jin G., Ma H. (2014). A genome-wide gene-environment interaction analysis for tobacco smoke and lung cancer susceptibility. Carcinogenesis.

[28] Thorgeirsson T.E., Stefansson K. (2010). Commentary: Gene-environment interactions and smoking-related cancers. Int. J. Epidemiology.

[29] VanderWeele T.J., Asomaning K., Tchetgen E.J.T., Han Y., Spitz M.R., Shete S., Wu X., Gaborieau V., Wang Y., McLaughlin J. (2012). Genetic Variants on 15q25.1, Smoking, and Lung Cancer: An Assessment of Mediation and Interaction. Am. J. Epidemiol..

[30] Maher B. (2008). Personal genomes: The case of the missing heritability. Nat. Cell Biol..

[31] Buttitta F., Barassi F., Fresu G., Felicioni L., Chella A., Paolizzi D., Lattanzio G., Salvatore S., Camplese P.P., Rosini S. (2006). Mutational analysis of theHER2 gene in lung tumors from Caucasian patients: Mutations are mainly present in adenocarcinomas with bronchioloalveolar features. Int. J. Cancer.

[32] Amos I.C., Wu X., Broderick P., Gorlov I.P., Gu J., Eisen T., Dong Q., Zhang Q., Gu X., Vijayakrishnan J. (2008). Genome-wide association scan of tag SNPs identifies a susceptibility locus for lung cancer at 15q25.1. Nat. Genet..

[33] Le Marchand L., Derby K.S., Murphy S.E., Hecht S.S., Hatsukami D., Carmella S.G., Tiirikainen M., Wang H. (2008). Smokers with the CHRNA Lung Cancer–Associated Variants Are Exposed to Higher Levels of Nicotine Equivalents and a Carcinogenic Tobacco-Specific Nitrosamine. Cancer Res..

[34] McKay J.D., Hung R.J., Gaborieau V., Boffetta P., Chabrier A., Byrnes G., Zaridze D., Mukeria A., Szeszenia-Dabrowska N., Lissowska J. (2008). Lung cancer susceptibility locus at 5p15.33. Nat. Genet..

[35] Landi M.T., Chatterjee N., Yu K., Goldin L.R., Goldstein A.M., Rotunno M., Mirabello L., Jacobs K., Wheeler W., Yeager M. (2009). A Genome-wide Association Study of Lung Cancer Identifies a Region of Chromosome 5p15 Associated with Risk for Adenocarcinoma. Am. J. Hum. Genet..

[36] Wang Y., Broderick P., Webb E., Wu X., Vijayakrishnan J., Matakidou A., Qureshi M., Dong Q., Gu X., Chen W.V. (2008). Common 5p15.33 and 6p21.33 variants influence lung cancer risk. Nat. Genet..

[37] Truong T., Hung R.J., Amos C.I., Wu X., Bickeböller H., Rosenberger A., Sauter W., Illig T., Wichmann H.-E., Risch A. (2010). Replication of Lung Cancer Susceptibility Loci at Chromosomes 15q25, 5p15, and 6p21: A Pooled Analysis From the International Lung Cancer Consortium. J. Natl. Cancer Inst..

[38] Miki D., Kubo M., Takahashi A., Yoon K.-A., Kim J., Lee G.K., Zo J.I., Lee J.S., Hosono N., Morizono T. (2010). Variation in TP63 is associated with lung adenocarcinoma susceptibility in Japanese and Korean populations. Nat. Genet..

[39] Li Y., Xiao X., Han Y., Gorlova O., Qian D., Leighl N., Johansen J.S., Barnett M., Chen C., Goodman G. (2017). Genome-wide interaction study of smoking behavior and non-small cell lung cancer risk in Caucasian population. Carcinogenesis.

[40] Wang J., Liu Q., Yuan S., Xie W., Liu Y., Xiang Y., Wu N., Wu L., Ma X., Cai T. (2017). Genetic predisposition to lung cancer: Comprehensive literature integration, meta-analysis, and multiple evidence assessment of candidate-gene association studies. Sci. Rep..

[41] Liao Y., Yang F., Li X., Chen K., Zhou L., Wang Y., Wang J. (2015). The impact of Caspase-8 on non-small cell lung cancer brain metastasis in II/III stage patient. Neoplasma.

[42] Camp N.J., Lin W., Bigelow A., Burghel G., Mosbruger T.L., Parry M.A., Waller R.G., Rigas S.H., Tai P.-Y., Berrett K. (2016). Discordant Haplotype Sequencing Identifies Functional Variants at the 2q33 Breast Cancer Risk Locus. Cancer Res..

[43] Shivapurkar N., Toyooka S., Eby M.T., Huang C.X., Sathyanarayana U.G., Cunningham H.T., Reddy J.L., Brambilla E., Takahashi T., Minna J.D. (2002). Differential inactivation of caspase-8 in lung cancers. Cancer Biol. Ther..

[44] Liu D., Xu W., Ding X., Yang Y., Lu Y., Fei K., Su B. (2017). Caspase 8 polymorphisms contribute to the prognosis of advanced lung adenocarcinoma patients after platinum-based chemotherapy. Cancer Biol. Ther..

