# Supplementary material for: Polymorphisms in the Gene Encoding Caspase 8 May Predict the Response to First-Line Platinum-Based Chemotherapy in Locally Advanced or Advanced Non-Small-Cell Lung Cancer
Source: J Clin Med. 2021 Mar 8;10(5):1126. doi: 10.3390/jcm10051126 (PMC7962636; doi:10.3390/jcm10051126)
Supplement: Supplementary file 1 [file jcm-10-01126-s001.pdf]

Supplementary Table 1. Comparisons of data distribution according to different study group sets.

Variable		Study group (n=99)	Patients included in the 1st evaluation of objective response (n=74)	*p* *(vs study group)*	Patients included in the 2nd evaluation of objective response (n=50)	*p* *(vs study group)*	Patients included in the survival analysis (n=89)	*p* *(vs study group)*
Gender	Male	76 (76.77%)	60 (81.08%)	0.5756	42 (84.00%)	0.3939	70 (78.65%)	0.8611
	Female	23 (23.23%)	14 (18.92%)		8 (16.00%)		19 (21.35%)	
Age	≥ 66.5	49 (49.50%)	32 (43.24%)	0.4442	19 (38.00%)	0.2236	44 (49.44%)	1.0000
	< 66.5	50 (50.50%)	42 (56.76%)		31 (62.00%)		45 (50.56%)	
Disease stage	III	35(35.35%)	28 (37.84%)	0.7519	21 (42.00%)	0.4758	29 (32.58%)	0.7585
	IVA	64 (64.65%)	46 (62.16%)		29 (58.00%)		60 (67.42%)	
Performance status (ECOG score)	0	17 (17.17)	11 (14.86%)	0.8352	9 (18.00%)	1.0000	8 (8.99%)	0.1320
	1 and 2	82 (82.83%)	63 (85.14%)		41 (82.00%)		81 (91.01%)	
Smoking status	Smoker	92 (92.93%)	69 (93.24%)	1.0000	47 (94.00%)	1.0000	84 (8.99%)	0.3408
	Non-smoker	7 (7.07%)	5 (6.76%)		3 (6.00%)		3 (91.01%)	
Pack years	≥ 45	45 (45.45%)	36 (48.65%)	0.7584	23 (46.00%)	1.0000	43 (48.31%)	0.7701
	< 45	54 (54.55%)	38 (51.35%)		27 (54.00%)		46 (51.69%)	
I line chemotherapy (I)	Cis + PEM	23 (23.23%)	17 (22.97%)	1.0000	13 (26.00%)	0.2818	22 (24.72%)	0.8849
	Other	76 (76.77%)	57 (77.03%)		26 (74.00%)		67 (75.28%)	
I line chemotherapy (II)	PN	60 (60.60%)	48 (64.86%)	0.6351	32 (64.00%)	0.7241	55 (61.80%)	0.8820
	Other	39 (49.40%)	26 (35.14%)		18 (36.00%)		34 (38.20%)	
I line chemotherapy (III)	PG	16 (16.16%)	7 (9.46%)	0.2593	3 (6.00%)	0.1172	13 (14.61%)	0.8411
	Other	83 (83.84%)	67 (90.54%)		47 (94.00%)		76 (85.39%)	
Distant metastases	No	43 (43.43%)	23 (31.02%)	0.1147	17 (34.00%)	0.2930	35 (39.32%)	0.6568
	Yes	56 (56.57%)	51 (68.92%)		33 (66.00%)		54 (60.68%)	
Weight	≥ 74	49 (49.49%)	34 (45.94%)	0.6485	27 (54.00%)	0.7287	43 (48.31%)	0.8849
	< 74	50 (50.51%)	40 (54.06%)		23 (46.00%)		46 (51.69%)	

BMI	≥ 24.91	49 (49.49%)	33 (44.59%)	0.5417	27 (54.00%)	0.7287	43 (48.31%)	0.8849
	<24.91	50 (50.51%)	41 (55.41%)		23 (46.00%)		46 (51.69%)	
Body loss (%)	≥ 9.84	32 (32.32%)	25 (33.78%)	0.8711	15 (30.00%)	0.8529	28 (31.46%)	1.0000
	< 9.84	67 (67.68%)	49 (66.22%)		35 (70.00%)		61 (68.54%)	
Time from diagnosis to treatment (days)	≥ 17	48 (48.48%)	25 (33.78%)	0.2618	25 (50.00%)	0.8643	44 (49.44%)	1.0000
	<17	51 (51.52%)	39 (66.22%)		25 (50.00%)		45 (50.56%)	
Occupational exposure	No	55 (79.71%)	55 (74.32%)	1.0000	38 (76.00%)	0.6583	50 (56.18%)	1.0000
	Yes	14 (20.29%)	14 (25.68%)		12 (24.00%)		13 (43.82%)	
Family history of malignancy (any)	No	46 (46.46%)	36 (48.65%)	0.8778	23 (46.00%)	1.0000	42 (47.19%)	1.0000
	Yes	53 (53.54%)	38 (51.35%)		27 (54.00%)		47 (52.81%)	
Family history of malignancy (lung)	No	75 (75.76%)	54 (72.97%)	0.7258	14 (28.00%)	0.0299*	68 (76.40%)	1.0000
	Yes	24 (24.24%)	20 (27.03%)		13 (72.00%)		21 (23.60%)	
Histopathology (I)	AC	40 (40.40%)	29 (39.19%)	0.8769	18 (36.00%)	0.7222	34 (38.20%)	0.7673
	SCC and NOS	59 (59.60%)	45 (60.81%)		32 (64.00%)		55 (61.80%)	
Histopathology (II)	SCC	52 (52.53%)	40 (54.05%)	0.8784	27 (54.00%)	1.0000	45 (50.56%)	0.8839
	AC and NOS	47 (47.47%)	34 (45.95%)		23 (46.00%)		44 (49.44%)	
CASP-8 (rs3769818)	AA	8 (8.08%)	7 (9.46%)	0.7895	3 (6.00%)	0.7514	7 (7.86%)	1.0000
	AG and GG	91 (91.92%)	67 (90.54%)		47 (94.00%)		82 (92.14%)	
CASP-8 (rs3769818)	AG	38 (38.38%)	28 (37.84%)	1.0000	19 (38.00%)	1.0000	25 (28.10%)	0.1641
	AA and GG	61 (61.62%)	46 (62.16%)		31 (62.00%)		64 (71.90%)	
CASP-8 (rs3769818)	GG	53 (55.21%)	39 (52.70%)	1.0000	24 (48.00%)	0.6032	57 (64.04%)	0.1821
	AG and AA	46 (44.79%)	35 (47.30%)		26 (52.00%)		32 (35.96%)	

Abbreviations: AC - adenocarcinoma; BMI - body mass index; Cis - Cisplatin; NOS - not otherwise specified; OR - odds ratio; SCC - squamous cell carcinoma; PEM - Pemetrexed; PG - Cisplatin+Gemcytabine PN - Cisplatin+Vinorelbine.

* - statistically significant result.
